# *Acinetobacter baumannii* strains isolated from patients in intensive care units in Goiânia, Brazil: Molecular and drug susceptibility profiles

**DOI:** 10.1371/journal.pone.0176790

**Published:** 2017-05-05

**Authors:** Suellen Rocha Araújo Castilho, Cássia Silva de Miranda Godoy, Adriana Oliveira Guilarde, Juliana Lamaro Cardoso, Maria Cláudia Porfirio André, Ana Paula Junqueira-Kipnis, André Kipnis

**Affiliations:** 1 Institute of Tropical Pathology and Public Health of Federal University of Goiás, Goiânia, Goiás, Brazil; 2 Hospital of Tropical Diseases Dr. Anuar Auad, Goiania, Goiás, Brazil; Ross University School of Veterinary Medicine, SAINT KITTS AND NEVIS

## Abstract

Resistance to antimicrobial agents is increasing worldwide and imposes significant life-threatening risks to several different populations, especially those in intensive care units (ICUs). Bacteria can quickly develop or acquire resistance to antimicrobial drugs, and combined with their intrinsic potential to cause disease in humans, these bacteria can become deadly. Among Gram-negative bacteria, *Acinetobacter baumannii* is notorious as a frequent opportunistic pathogen associated with critically ill patients, and understanding the genetic basis of *A*. *baumannii* resistance to beta-lactams among patients in ICUs will result in better protocols to prevent the development of resistance as well as improved treatment regimens. In this study, we assessed 1333 patients in five ICUs, 56 of whom developed *A*. *baumannii* infections. Most of the *A*. *baumannii* isolates were resistant to beta-lactam antimicrobial drugs, specifically, 3^rd^- and 4^th^-generation cephalosporins and carbapenems, and 91.1% of the isolates were multi-drug resistant (MDR). The most frequent OXA gene present was OXA-23 (55.1%), which is significantly associated with MDR strains. Most of the *A*. *baumannii* isolates (76.8%) were capable of forming a biofilm. The antimicrobial drug classes that were effective against most of these isolates were polymyxins and tigecycline. The molecular profile of the isolates allowed detection of 12 different clusters comprising 2 to 8 isolates each. In conclusion, our data indicate a high incidence of resistance to carbapenems as well as MDR strains among the observed *A*. *baumannii* isolates, most of which exhibited a high prevalence of OXA-23 gene expression. Only a few selective drugs were effective, reinforcing the notion that bacterial resistance is an emerging problem that should be prioritized in every healthcare facility.

## Introduction

In the past three decades, *Acinetobacter baumannii* has emerged as a major opportunistic infectious pathogen in critically ill patients who have serious underlying disease, have been hospitalized for long periods, and/or are undergoing invasive procedures with prior use of antimicrobial drugs. This bacterium plays a significant role in healthcare-associated infections (HAI) in institutions worldwide, especially in adult intensive care units (ICUs) [1].

Worldwide bacterial resistance to antimicrobial drugs has caused the United Nations to put forth a global effort to control antimicrobial resistance [2]. Emergence of multi-drug-resistant (MDR) *A*. *baumannii* strains causing nosocomial infections has afflicted various countries in Europe, Asia, Latin America and other continents where global outbreaks have been described [3]. The majority of MDR cases have occurred in adults attending intensive healthcare units [4–8], and resistance to carbapenems is the most common phenotype [5].

In Latin America, the Antimicrobial Surveillance Program (SENTRY) examined susceptibility to antimicrobial drugs among 826 *Acinetobacter* spp. isolates in seven countries between 1997 and 2001, with the greatest number of isolates originating from Brazil. A gradual decline in carbapenem susceptibility was observed during the study period [9]. Furthermore, in Brazil, a country of continental proportions with broad geographical and economic diversity and a population of 192 million, *A*. *baumannii* has emerged as a major nosocomial pathogen, causing infections in ICUs with numerous outbreaks, especially involving isolates resistant to carbapenems [10].

*A*. *baumannii* presents resistance to carbapenems by acquiring genes that encode carbapenem-degrading enzymes, interfere with drug permeability and/or alter antimicrobial target affinity. Among these enzymes, Ambler class B metalloenzymes and class D oxacillinases are the most common participating in resistance mechanisms [11]. The former are powerful carbapenemases, among which OXA-type β-lactamases are the most prevalent type [1,11]. Although more than 250 OXA types have been described worldwide, four carbapenemase groups are highlighted due to their frequency and importance: OXA-23-like, OXA-24-like, OXA-58-like, and OXA-51-like [12]. The OXA-23 subgroup has been detected in several countries on all continents, including Brazil [13–15]. Resistance to carbapenem increases significantly when OXA genes are juxtaposed next to insertion elements with promoter functions, the most effective of which is the insertion sequence element of *A*. *baumannii* (IS*Aba1*) [11,16,17]. Biofilm formation is another important feature of *A*. *baumannii* that is associated with infection, and this characteristic is also related to antimicrobial drug resistance [18].

The incidence of *A*. *baumannii* nosocomial infection has increased considerably in Brazil in recent years [9]. In Goiânia, the capital of Goiás in the central region of Brazil, healthcare is provided not only to its inhabitants but also to the population of many cities in this region and the northwestern Brazil. Thus, studying nosocomial infections caused by non-fermenting Gram-negative bacilli (NF-GNB) in this region is of utmost importance.

An ICU is the healthcare department where the most seriously ill patients are attended, and *A*. *baumannii* infections in these patients impose life-threatening risks. Although a matter of debate, resistance to different drugs, including carbapenems, has contributed to increases in mortality rates [19]. The aim of the present study was to evaluate the susceptibility, biofilm formation, and genetic profiles of *A*. *baumannii* isolates from patients from different ICUs and to correlate the MDR phenotype of these isolates with the ability to form a biofilm.

## Materials and methods

Experimental Design: a descriptive cohort of adults (≥ 18 years) from five ten-bed intensive care referral units in the Brazilian state of Goiás who were infected with *A*. *baumannii* between June and December 2010 were included in this study. Individuals positive for infection less than 48 hours after admission were excluded.

### Bacterial isolation, identification and drug susceptibility testing

The collection of biological material was conducted according to physicians’ recommendations. The samples were sent to the local referral laboratory for *A*. *baumannii* isolation and identification using manual and/or automated methods (Vitek II, bioMérieux, Marcy l’Etoile, France). Bacteria were most commonly obtained from tracheal aspirate (44.0%), blood (20.2%), or central venous catheter (16.6%) samples.

Bacterial isolates were identified by Gram staining and the following properties: motility, growth at 42°C, citrate utilization, oxidase and urease production, oxidative/fermentation (OF)-glucose test, bile esculin hydrolysis test, decarboxylation of amino acids (i.e., lysine, ornithine and arginine) and OF-lactose test at 10%. In two ICUs, an automated system (Vitek II) was used for isolate identification. Additionally, polymerase chain reaction (PCR) to amplify the OXA51 gene was performed on all presumptively identified *A*. *baumannii* isolates [17,20]. Drug susceptibility testing was carried out using the disc diffusion method according to the Clinical and Laboratorial Standards Institute for the following drugs: ampicillin/sulbactam, amikacin, gentamicin, ceftazidime, cefepime, ciprofloxacin, levofloxacin, imipenem, meropenem, piperacillin/tazobactam, polymyxin B/colistin, tetracycline, and tigecycline. The E-test was performed for polymyxin susceptibility testing (resistance was considered at a minimum inhibitory concentration (MIC) ≥ 4 μg/mL). Multi-drug resistance was defined as resistance to three or more classes of the drugs tested [21].

### DNA extraction and gene amplification by PCR

Cultures were grown on TSA (tryptic soy agar) and subcultured in TSB (tryptic soy broth) for DNA extraction. Chromosomal DNA was extracted based on a method described by van Soolingen et al. [22].

The primers used in this study for blaOXA-23, blaOXA-40, blaOXA-51, blaOXA-58, and IS*Aba1* were described previously [16]; the sequences are presented in Table 1. PCR was performed in a final volume of 20 μL containing 20 ng DNA, 1.5 mM MgCl_2_, 250 μM each dNTP, 1 U Taq DNA Polymerase (Promega, Madison, WI, USA), and 0.47 μM each primer using a thermal cycler (Biocycler MJ96G; Applied Biosystems, Foster City, USA) under the following conditions: denaturation at 95°C for 4 min; 35 cycles of denaturation (90°C, 30 sec), annealing (according to the target gene, as listed in Table 1, 40 sec) and extension (72°C, 1 min); and a final extension step at 72°C for 5 min. DNA samples resulting in the amplification of any of the tested OXA genes were investigated for the presence of the IS*Aba1* element upstream of each OXA gene. For that purpose, downstream primers for each OXA gene were combined with the forward primer for IS*Aba1* in a new PCR reaction. PCR products were separated by 1.5% agarose gel electrophoresis, stained with 0.5 μg/mL ethidium bromide and visualized using Gel Doc System XR (Bio-Rad, Laboratories, Hercules, CA, USA).

**Table 1:** Primer sequences used for detection of OXA type β-lactamases and IS*Aba1* in *Acinetobacter baumannii* isolates.

Primers	Sequence (5’—3’)	Product size (bp)	Tm*
OXA-23 F	GATGTGTCATAGTATTCGTCGT	1,057	50
OXA-23 R	TCACAACAACTAAAAGCACTGT		
OXA-40 F	ATGAAAAAATTTATACTTCCTATATTCAGC	825	50
OXA-40 R	TTAAATGATTCCAAGATTTTCTAGC		
OXA-51 F	AACAAGCGCTATTTTTATTTCAG	641	50
OXA-51 R	CCCATCCCCAACCACTTTT		
OXA-58 F	AGTATTGGGGCTTGTGCT	453	49
OXA-58 R	AACTTCCGTGCCTATTTG		
IS*Aba1* F	CATTGGCATTAAACTGAGGAGAAA	451	50
IS*Aba1* R	TTGGAAATGGGGAAAACGAA		

* Tm: melting temperature (°C)

### Biofilm formation

Biofilm formation was estimated quantitatively according to the method described by Tendolkar et al. [23], with some modifications. The *A*. *baumannii* strain ATCC 19606 and *Escherichia coli* HB 101 were used as a positive and negative control, respectively. After *A*. *baumannii* growth in TSB for 24 hours at 37°C, the culture concentration was adjusted to 0.5 based on the McFarland scale and diluted 10 times in Luria Bertani (LB) broth at 1/4^th^ of its concentration with 0.2% glucose (LB¼-Gli). A 300-μL aliquot of the adjusted culture was dispensed into a 96-well culture plate and incubated for 22 hours at 29°C. Culture growth was determined by measuring the absorbance at 405 nm. Biofilms were stained with 0.2% (w/v) crystal violet and quantified at 595 nm after solubilization with ethanol/acetone (80:20 v/v). Biofilm quantifications were then corrected to the culture growth (ratio of the optical density at 595 nm to the optical density at 405 nm). Experiments were conducted in four replicates and independently repeated three times. Samples with OD ratios that were significantly higher (*t* test) than the negative control were considered positive for biofilm formation.

### Molecular typing

Genetic similarities among the *A*. *baumannii* isolates were investigated by pulsed-field gel electrophoresis (PFGE). The *A*. *baumannii* isolates were sent to the Molecular Biology Laboratory at the Tropical Pathology and Public Health Institute (IPTSP) where preparation of genomic DNA was performed as described by Seifert et al. [24]. The bacterial suspension was digested with the restriction enzyme *Apa*I, and the DNA fragments were separated by 1% agarose gel electrophoresis for 19 hours using a CHEF-DR II apparatus (Bio-Rad), with pulses varying from 5–20 s at a voltage of 6 V/cm. After staining with ethidium bromide (0.5 μg/mL), the resulting fragments were examined using BioNumerics v. 5.1 software (Applied Maths, Sint-Martens-Latem, Belgium) [24]. Clustering was performed using Unweighted Pair Group Method with Arithmetic averages (UPGMA). Similarity among the isolates was estimated using the Dice correlation coefficient with 0.7% optimization and a 1.0% tolerance setting. For clustering of the isolates, a cut-off of 80% was applied. Identical PFGE profiles (100% similarity) were defined as a pulsotype (PT).

### Data processing and analysis

Descriptive analysis of the demographic characteristics of the patients was performed. Measures of central tendency and dispersion were calculated for continuous variables, and frequencies were calculated for categorical variables. The χ^2^ and Fisher exact tests were used to compare categorical variables when necessary. Analysis of variance was used for continuous variables. The level of significance was set at p<0.05. The Statistical Package for Social Science (SPSS/PC-16.0) was used for all analyses.

Ethical considerations: The study was performed in accordance with the principles expressed in the Declaration of Helsinki and was approved by the Research Ethics Committees from the participating institutions (Protocols: CEP ACCG No. 005/10 from the Ethics Committee of the Araújo Jorge Hospital at Goiania, Goiás; HC/UFG No. 070/2010 from Ethics Committee of the Federal University of Goiás and HDT No. 002/2010 from the Ethics Committee of the Hospital of Tropical Diseases Dr. Anuar Auad at Goiania, Goiás). The study was described to all participating individuals or their legally authorized representatives, and informed consent was signed by all.

## Results

During the six-month period covered by the study, 1333 patients were admitted to the five ICUs included. Of these, 64 patients infected with *A*. *baumannii* were enrolled, and 84 *A*. *baumannii* isolates were obtained, corresponding to a 4.8% frequency of *A*. *baumannii* infection. Among the 64 patients, 56 (87.5%) were considered cases of infection with clinical symptoms and 6 (9.4%) cases of colonization. The mean age of the participants was 53.2 years (sd = 19 years), and 59.4% were male. The median length of the overall hospital stay of the patients was 32.5 days [interquartile interval (IQR): 17.2–45.0], and the median length of stay in the ICU was 15.5 days (IQR: 7.0–33.2). Most patients (98.4%) had previously taken antimicrobials, and 68.2% had used two or more classes of antimicrobial drugs. The most commonly used drugs were 3^rd-^ and 4^th^-generation cephalosporins (71.4%) and carbapenems (50.8%). There was no significant difference among patients at the different ICUs in terms of time until acquisition of infection, previous use of antimicrobials, resistance to carbapenems, adequate initial antimicrobial therapy or mortality.

The most frequent underlying diseases found were neoplasia (34.4%), acquired immune deficiency syndrome (AIDS) (17.2%), diseases of the digestive tract (14.1%), cardiovascular disorders (7.8%), respiratory disease (6.2%), chronic kidney failure (6.2%), and neuropathies (3.1%). In terms of the evaluated severity of underlying diseases upon admission to the ICU, 64.1% were defined as having a fatal illness and 34.4% a life-threatening condition.

Among the different invasive procedures conducted prior to detecting *A*. *baumannii* infection, the most frequent was insertion of a central vascular catheter (CVC) probe, at 93.7% (60). The mean length of time that patients had a CVC was 14.8 days (sd = 10.5); for a delayed vesicle probe (DVP), the mean length of time was 14.1 days (sd = 10.8). A total of 53 patients (82.8%) underwent endotracheal intubation and mechanical ventilation for a mean duration of 13.1 days (sd = 11.1).

In terms of the topography of infection, the lungs were the most common site (53.1%), followed by the site of surgical intervention (10.9%), the urinary tract (7.8%) and the blood stream (i.e., sepsis) (6.2%). The overall mortality was 79.7% (51/64), and mortality relating to *A*. *baumannii* infections was 67.9% (38/56). The evaluation of possible prognostic factors for determining mortality in the 38 patients who died excluded death from other causes and patients who were only colonized with *A*. *baumannii*. One isolate per patient (56 patients) was available for culturing and molecular analysis.

Antibiotic susceptibility profiling of *A*. *baumannii* isolates obtained from patients in a hospital setting is of utmost importance to guide clinicians in providing the most appropriate drug therapy. The incidence of *A*. *baumannii* strains resistant to carbapenems was 76.8%. Resistance to the other tested antimicrobials was as follows: ampicillin/sulbactam, 60.7%; cefepime, 96.4%; quinolones, 91.1%; amikacin, 21.4%; polymyxin B, 8.9%; and tigecycline, 7.1%. One patient was infected with an isolate that exhibited resistance to all tested antimicrobial drugs (pandrug resistant, PDR). Fifty-one of the isolates (91.1%) were MDR, including 4 isolates categorized as extensively drug resistant (XDR).

Several OXA gene types have been associated with drug resistance profiles among *A*. *baumannii* strains isolated in hospital settings. To comprehend the molecular basis of the drug resistance of the isolates, the presence of OXA genes 23, 40, 51 and 58 was evaluated. None of the isolates presented OXA-40; in contrast, 55.1% contained OXA-23, and 3.6% harbored OXA-58. The presence of the OXA-23 gene was significantly associated with the MDR phenotype, though such an association was not found with regard to OXA-58 (Table 2).

**Table 2:** Association between OXA gene types and multi-drug resistance.

	Not MDR	MDR*	p value**
OXA-23 present	3	29	0.002
OXA-23 absent	12	12	
OXA-58 present	0	2	1
OXA-58 absent	15	39	
OXA-40 present	0	0	NA
OXA-51 present	15	41	NA

* MDR: cefepime, ceftazidime, ciprofloxacin, imipenem and meropenem.

** Fisher’s exact test. NA, not applicable.

The presence of insertion elements upstream of some OXA genes may increase the bacterial resistance spectrum to antimicrobials, with IS*Aba1* as one of the most important elements. Hence, we searched for the IS*Aba1* insertion element upstream of the identified OXA genes (Table 3). When present, the OXA23 gene was associated with the IS*Aba1* element at a frequency of 90.2% (29/32), accounting for 51.8% of all obtained *A*. *baumannii* isolates in this study. Most of the isolates were OXA23 positive and MDR, with an IS*Aba1*/OXA23 association rate of 62.1% (18/29). Association of IS*Aba1*/OXA-51 was detected in 30.3% of the isolates, but in contrast to OXA23, most isolates that presented the IS*Aba1*/OXA-51 association were not MDR (2/17, 11.8%). Although both isolates that presented the OXA 58 gene were MDR, the IS*Aba1* element was not found in either.

**Table 3:** Association between the presence of the IS*Aba*1 element and multi-drug resistance.

	Not MDR*	MDR
IS*Aba1*/OXA-23	3	26
OXA-23 without IS*Aba1*	0	3
IS*Aba1*/OXA-51	4	13
OXA-51	11	28

* MDR: cefepime, ceftazidime, ciprofloxacin, imipenem and meropenem

The susceptibility profile of the *A*. *baumannii* isolates to beta-lactam antimicrobials with regard to the presence of OXA genes was also investigated (Table 4). Regardless of the observed antimicrobial drug resistance, the most prevalent genetic profile was IS*Aba1*/OXA-23 (20 isolates with resistance to at least one of the tested drugs), followed by the presence of both IS*Aba1*/OXA-23 and IS*Aba1*/OXA-51 (9 isolates).

**Table 4:** OXA gene frequencies according to drug resistance profiles.

Antimicrobial resistance profile	A* (n = 1)	B (n = 20)	C (n = 1)	D (n = 5)	E (n = 2)	F (n = 9)	G (n = 17)
Cefepime	1	20	1	5	2	9	15
Ceftazidime	1	20	1	5	2	9	13
Imipenem	1	19	1	2	2	9	8
Meropenem	1	19	1	2	2	9	8
Ampicillin/Sulbactam	0	14	0	2	2	8	7
All beta-lactams	0	14	0	2	2	8	6

* **A:** OXA-23; **B**: IS*Aba1*/OXA-23; **C**: OXA-58; **D**: IS*Aba1*/OXA-51; **E**: IS*Aba1*/OXA-51+OXA-23; **F**: IS*Aba1*/OXA-51+IS*Aba1*/OXA-23; **G**: OXA-51 only.

Biofilm formation is a frequent characteristic of *A*. *baumannii* isolates. In our study, we found that 76.8% (43) of the isolates were capable of forming biofilm, and most of the isolates capable of forming biofilm (40 isolates, 93%) were also MDR. Nonetheless, a high percentage of isolates that did not form a biofilm were also MDR (11 of 13 isolates); consequently, no association between biofilm formation and MDR was observed (Fischer’s exact test p = 0.58). Most of the biofilm-forming isolates (95.3%, 41 of 43) presented resistance to at least one tested antimicrobial drug. In contrast, as shown in Table 5, the non-biofilm forming isolates were less resistant to the different drug classes. Resistance to polymyxin B/colistin and tigecycline was observed in three isolates capable of biofilm formation and one isolate incapable of biofilm formation. One additional isolate that formed biofilm was resistant to polymyxin and susceptible to tigecycline. All four isolates that were resistant to polymyxin were also MDR, including resistance to imipenem.

**Table 5:** Biofilm formation among drug-resistant *A*. *baumannii* isolates.

Antimicrobial drug	Resistant isolates (%), n = 56	Biofilm positive (%)	Biofilm negative (%)
Cefepime	54 (96.4)	41 (75.9)	13 (24.1)
Ceftazidime	52 (92.8)	39 (75.0)	13 (25.0)
Ciprofloxacin	51 (91.0)	39 (76.4)	12 (23.6)
Imipenem	43 (76.7)	32 (74.4)	11 (25.6)
Meropenem	43 (76.7)	32 (74.4)	11 (25.6)
Piperacillin / Tazobactam	39 (69.6)	31 (79.5)	8 (20.5)
Ampicillin / Sulbactam	34 (60.7)	28 (82.3)	6 (17.7)
Gentamicin	32 (57.1)	26 (81.2)	6 (18.8)
Tetracycline	24 (42.8)	19 (79.1)	5 (20.9)
Levofloxacin	20 (35.7)	16 (80.0)	4 (20.0)
Amikacin	12 (21.4)	10 (83.3)	2 (16.7)
Polymyxin B / Colistin	5 (60.7)	4 (80.0)	1 (20.0)
Tigecycline	4 (7.1)	3 (75.0)	1 (25.0)

**Fig 1:**
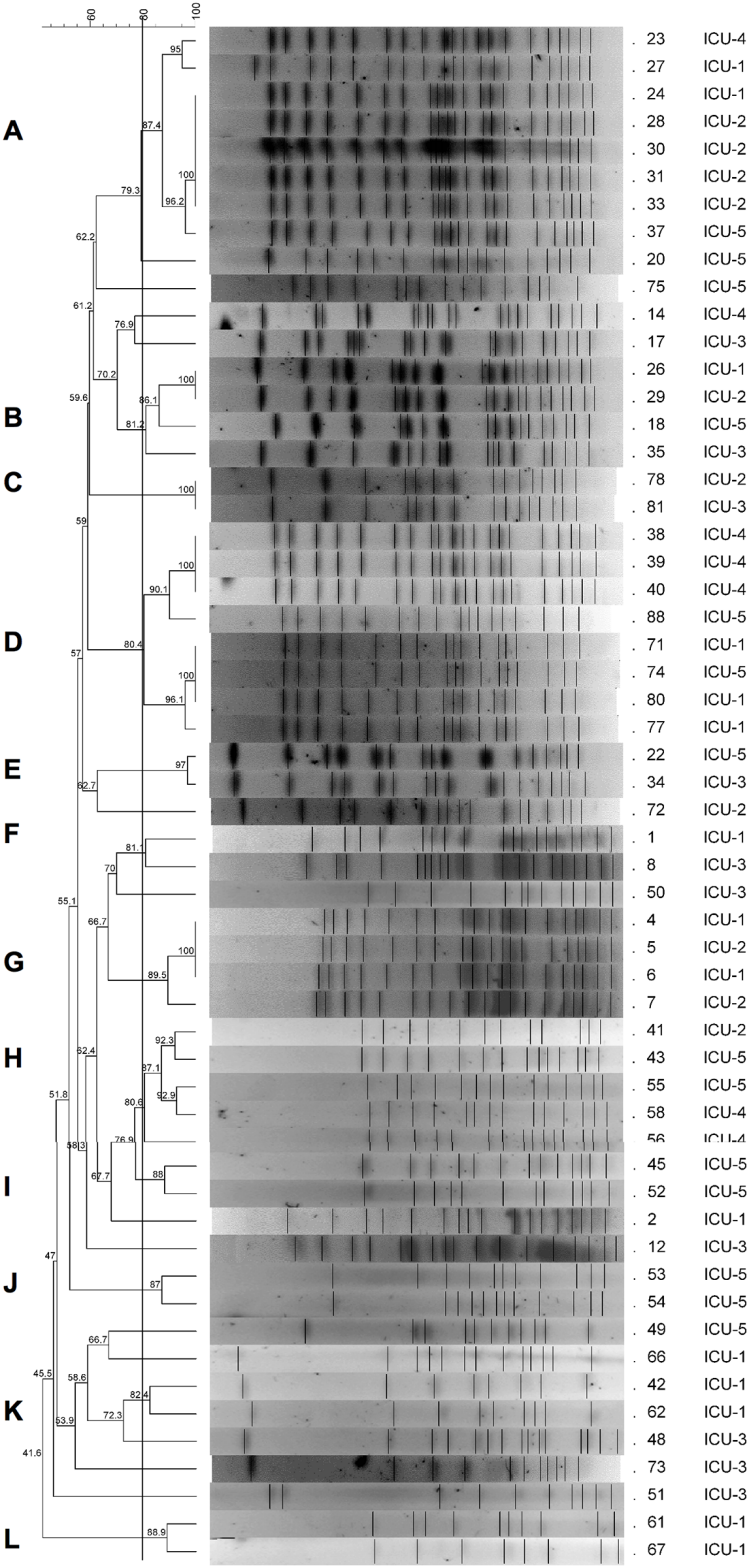
Similarity among *Acinetobacter baumannii* strains studied. Dendrogram representing PFGE profiles of *Acinetobacter baumannii* strains isolated from 56 patients from five different ICUs in Goiânia, Brazil. The cut-off point was set at 80% for the similarity coefficient (vertical line). Twelve different clusters (A thru L at the left) were detected. The identification number of the isolates and the ICUs can be found to the right of the profiles.

MDR *A*. *baumannii* clusters have been associated with hospital-acquired infections, and identification of isolates within these clusters can promote the adoption of control measures by the infection control team. We performed PFGE analysis for all 56 isolates (one per patient) from the different ICUs, and several isolates grouped into the same cluster (Fig 1), with 43 isolates included in twelve clusters (containing 2 to 8 isolates each). Eight clusters grouped isolates obtained from different ICUs (Clusters A thru H). Cluster A grouped 8 isolates resistant to carbapenems from patients at four different ICUs. Detection of the first isolate in this cluster occurred in June 2010 at ICU 1, and the same clone was isolated in four other patients from ICU 2 in the same hospital. Later, other closely related isolates were detected in ICUs 4 and 5 until September of the same year.

Of the 56 *A*. *baumannii* isolates analyzed, 43 showed resistance to carbapenems. All of the clusters contained isolates with the same carbapenem resistance profile for resistance or susceptibility to carbapenem, except for Clusters D and J, which contained both carbapenem-resistant and carbapenem-susceptible isolates (Fig 1). The five samples that showed resistance to polymyxin B did not have similar PFGE profiles (isolates 8, 12, 17, 18 and 35).

## Discussion

*Acinetobacter* infections and resistance development are well-known problems that are becoming increasingly more frequent and that must be adequately addressed throughout the world. Biofilm formation is an important virulence characteristic, especially among disseminated and mechanical-assisted ventilation-related bacterial diseases [25]. Here, we report a high rate of MDR strains among *A*. *baumannii* isolates from patients in ICUs. The cause of the high incidence of MDR strains in ICUs may be due to the excessive use of antimicrobials in the study population. In addition to the overall high rate of MDR strains circulating in our geographical region, our results show that isolates that are able to form biofilms are also strongly associated with multi-drug resistance. Furthermore, the majority of MDR *A*. *baumannii* strains harbored the OXA23 gene associated with the IS*Aba1* element.

Polymyxins are an alternative drug for the treatment of gram-negative bacteria [26]. Our results showed only six isolates (8.9%) to be resistant to polymyxin, supporting the possible use of this drug for treating *A*. *baumannii* infections in Brazil, as previously reported [27]. Similarly, relatively low levels of resistance (7.1%) to tigecycline, a drug used to treat *A*. *baumannii* strains that produce carbapenemases [28], were found in this study. Thus, tigecycline might serve as an alternative drug for treating this subgroup of bacteria, though its implementation either alone or in combination with other antibiotics remains a matter of debate [29]. Regardless, all of the isolates that were resistant to tigecycline were also resistant to polymyxin.

Several reports have suggested a correlation between the observed susceptibility of clinical isolates to the combination of ampicillin and sulbactam (AMS) and a successful approach to treating *A*. *baumannii* infections, particularly in cases in which resistance to carbapenems is present [30]. However, it has been recently shown that resistance to AMS among *A*. *baumannii* strains is increasing. Our study also reports this tendency, with a significantly high rate of resistance to AMS (60.7%), posing another challenge to adequate treatment of infections with these agents [31].

As genetic evaluation of bacterial strains is a time-consuming task, identifying appropriate genetic traits that could be related to the resistance profile could significantly reduce infection times and improve the characterization of clinical *A*. *baumannii* strains. The high frequency of OXA-23 observed among the studied isolates is in agreement with the literature. The association of the insertion element IS*Aba1* upstream of the OXA-23 and/or OXA-51 genes has been shown to be associated with multi-drug resistance, including strains isolated in Brazil [17,32]. However, we did not find such an association, which may be due to the high prevalence of MDR strains in the present study. We also report the emergence of *A*. *baumannii* isolates harboring the OXA-58 gene in the central region of Brazil. To the best of our knowledge, this is the first report of OXA 58 gene isolation from *A*. *baumannii* strains in central Brazil (GenBank accession numbers: KT148593.1 and KT148594.1). The appearance of OXA-58-positive strains has also been reported in other regions of Brazil, with similar low frequencies [33].

The insertion element IS*Aba1* is the most important factor associated with increased expression of OXA genes, and overexpression confers a more pronounced resistance profile [11,16,17]. Amplification of the IS*Aba1* sequence upstream of OXA genes in our study was detected among 82% of the isolates. A similarly high prevalence was also observed in isolates from other countries such as Spain (74.7%), the United Kingdom (84%) and Iran (90%) [16,17,34], demonstrating the high fitness of association between these genetic elements.

Among the *A*. *baumannii* isolates, 43 of 56 (76.8%) exhibited the ability to form biofilm. This is in agreement with other reported studies in which a rate of biofilm formation ranging from 55 to 75% was observed [35–37]. Although we did not investigate the environmental source of *A*. *baumannii* strains, due to the high frequency of biofilm-forming isolates encountered, identifying the sources of these strains should be addressed in the future. Approximately 71% of the isolates were MDR and capable of forming biofilm, supporting the close correlation between these characteristics. The formation of biofilm has been associated with the presence of the blaPER-1 gene [38], but no such association was observed in the strains analyzed in our study (data not shown).

The PFGE results showed that related strains were distributed throughout the ICUs investigated in this study and that outbreaks were detected during the study period. The presence of clusters with few isolates (Cluster 2 or 3) in the same ICU suggests the cross-transmission of *Ab* among patients in the unit, either by the healthcare team or by contaminated equipment and fomites, as described previously [39,40].

Transmission of closely related isolates from one ICU to another, within the same hospital or among ICUs from different hospitals within a short period of time highlights the transmission of strains from colonized or infected patients via patient transfer between two ICUs and the healthcare team within a facility or working in different ICUs or health institutions within the same city. This type of transmission has been described in several countries (including Brazil) and must be further investigated to establish better control measures [39–41].

An outbreak with a large number of isolates all resistant to carbapenems was detected by PFGE and affected eight patients at four of the five ICUs included in our study. It may be that the outbreak involved a large number of cases, as the first patient who tested positive for infection was the first case included in our study, and it is possible that more cases had occurred prior to the period covered by this study.

A common carbapenem resistance pattern was observed among all of the possibly related isolates grouped into clusters, with the exception of Clusters D and J. However, comparison of PFGE patterns of the isolates showing polymyxin B resistance did not reveal any clonal similarity among them. This result demonstrates the low discriminatory power of the antibiogram and emphasizes the need for molecular techniques such as PFGE to discriminate isolates with similar phenotypes but distinct genetic relatedness during evaluation of outbreak episodes or horizontal transmission in a hospital environment.

In this work, identification of *A*. *baumannii* strains was performed by biochemical and OXA-51 gene detection tests, which may not have appropriately discriminated all species from the *A*. *baumannii/calcoaceticus* complex [42]. Nonetheless, several authors have used this approach, without interference with the analysis and conclusions.

In conclusion, our data indicate that resistance to carbapenems as well as multi-drug resistance is common among *A*. *baumannii* isolates, with a high prevalence of the OXA-23 gene. Our data indicate that carbapenem resistance and multi-drug resistance are highly prevalent among *A*. *baumannii* strains isolated from ICU patients and that those strains possess the OXA-23 gene and the ability to form a biofilm.

## Supporting information

S1 File Supporting data for Tables 2–5. Individual sample data regarding biofilm formation capability, presence of antimicrobial resistance genes and susceptibility profiles.
(PDF)
