# Supplementary material for: Acinetobacter baumannii strains isolated from patients in intensive care units in Goiânia, Brazil: Molecular and drug susceptibility profiles
Source: PLoS One. 2017 May 5;12(5):e0176790. doi: 10.1371/journal.pone.0176790 (PMC5419545; doi:10.1371/journal.pone.0176790)
Supplement: S1 File — Individual sample data regarding biofilm formation capability, presence of antimicrobial resistance genes and susceptibility profiles. (PDF) [file pone.0176790.s001.pdf]

										Antimicrobial susceptibility test (R = resistant, S = susceptible, NT - not tested													
	Site of sample collection	Biofilm	OXA-23	ISAba1/OXA-23	OXA-40	OXA-51	ISAba1/OXA-51	OXA-58	ISAba1/OXA-58	AMP/ SUL	PIP/ TAZ	CFPM	CAZ	IMP	MER	AMI	GEN	CPX	LVX	POL	TET	TGC	Death
ABs01	Peritoneal fluid	Yes	Yes	Yes	No	Yes	Yes	No	No	R	R	R	R	R	R	I	R	R	NT	S	NT	S	Y
ABs02	Respiratory secretion	Yes	No	No	No	Yes	No	No	No	R	R	R	R	R	R	I	R	R	NT	S	NT	S	Y
ABs04	Respiratory secretion	Yes	Yes	No	No	Yes	Yes	No	No	R	R	R	R	R	R	S	R	R	NT	S	NT	S	N
ABs05	Catheter	Yes	Yes	Yes	No	Yes	No	No	No	R	R	R	R	R	R	S	S	R	NT	S	NT	S	N
ABs06	Respiratory secretion	Yes	Yes	Yes	No	Yes	Yes	No	No	R	R	R	R	R	R	S	R	NT	NT	S	NT	S	Y
ABs07	Pleural fluid	Yes	Yes	Yes	No	Yes	Yes	No	No	R	R	R	R	R	R	S	S	R	NT	S	NT	S	Y
ABs09	Blood	Yes	No	No	No	Yes	Yes	No	No	R	R	R	R	R	R	R	R	R	R	R	R	R	Y
ABs12	Catheter	Yes	Yes	No	No	Yes	Yes	No	No	R	R	R	R	R	R	S	R	R	R	R	R	R	Y
ABs14	Blood	Yes	No	No	No	Yes	No	No	No	NT	NT	R	R	S	S	R	R	R	NT	S	R	S	Y
ABs17	Urine	Yes	Yes	Yes	No	Yes	No	No	No	R	R	R	R	R	R	R	R	R	R	R	R	R	Y
ABs18	Blood	Yes	Yes	Yes	No	Yes	No	No	No	R	R	R	R	R	R	R	R	R	R	R	R	S	N
ABs20	Blood	Yes	No	No	No	Yes	No	No	No	R	R	R	R	R	R	S	S	R	R	S	R	S	Y
ABs22	Urine	Yes	No	No	No	Yes	No	No	No	S	S	R	R	S	S	S	R	R	R	S	R	S	Y
ABs23	Respiratory secretion	Yes	Yes	Yes	No	Yes	No	No	No	NT	NT	R	R	R	R	S	S	R	NT	S	R	S	Y
ABs24	Respiratory secretion	Yes	Yes	Yes	No	Yes	Yes	No	No	R	R	R	R	R	R	S	R	R	NT	S	NT	S	Y
ABs26	Respiratory secretion	Yes	No	No	No	Yes	No	No	No	R	R	R	R	R	R	S	R	R	NT	S	NT	S	Y
ABs27	Respiratory secretion	Yes	No	No	No	Yes	No	No	No	R	R	R	R	R	R	S	S	R	NT	S	NT	S	Y
ABs28	Urine	Yes	Yes	Yes	No	Yes	Yes	No	No	R	R	R	R	R	R	S	S	R	R	S	NT	S	Y
ABs29	Skin	Yes	Yes	Yes	No	Yes	No	No	No	R	R	R	R	R	R	I	R	R	NT	S	NT	S	N
ABs30	Respiratory secretion	Yes	Yes	Yes	No	Yes	Yes	No	No	R	R	R	R	R	R	S	R	R	NT	S	NT	S	Y
ABs31	Peritoneal fluid	Yes	Yes	Yes	No	Yes	Yes	No	No	R	R	R	R	R	R	S	R	R	NT	S	NT	S	Y
ABs33	Respiratory secretion	No	Yes	Yes	No	Yes	No	No	No	R	R	R	R	R	R	S	R	R	NT	S	NT	S	Y
ABs34	Blood	Yes	Yes	Yes	No	Yes	No	No	No	S	R	R	R	S	S	R	R	R	R	S	R	S	Y
ABs35	Blood	No	Yes	Yes	No	Yes	No	No	No	S	R	R	R	R	R	S	R	R	R	R	R	R	Y
ABs37	Catheter	No	Yes	Yes	No	Yes	No	No	No	R	R	R	R	R	R	R	S	R	I	S	NT	S	Y
ABs38	Respiratory secretion	Yes	Yes	No	No	Yes	No	No	No	NT	NT	R	R	R	R	S	S	R	NT	S	S	S	Y
ABs39	Blood	No	No	No	No	Yes	No	No	No	NT	NT	R	R	R	R	S	S	R	NT	S	S	S	Y
ABs40	Respiratory secretion	No	Yes	Yes	No	Yes	No	No	No	NT	NT	R	R	R	R	S	S	NT	NT	S	S	S	N
ABs41	secreção ferida operató	Yes	Yes	Yes	No	Yes	No	No	No	R	R	R	R	R	R	S	R	R	NT	S	NT	S	N
ABs42	Blood	Yes	No	No	No	Yes	No	No	No	R	R	R	R	S	S	S	S	R	NT	NT	NT	NT	Y
ABs43	Respiratory secretion	No	Yes	Yes	No	Yes	No	No	No	R	R	R	R	R	R	R	R	R	I	S	R	S	Y
ABs45	Respiratory secretion	Yes	Yes	Yes	No	Yes	No	No	No	R	R	R	R	R	R	S	S	R	R	S	R	S	Y
ABs48	Respiratory secretion	Yes	No	No	No	Yes	No	No	No	S	S	R	R	S	S	R	R	R	R	S	R	S	Y
ABs49	Catheter	Yes	No	No	No	Yes	Yes	No	No	S	R	R	R	S	S	S	S	R	R	S	R	S	Y
ABs50	Respiratory secretion	Yes	No	No	No	Yes	No	No	No	S	S	R	S	S	S	S	S	S	S	S	R	S	N
ABs51	Catheter	Yes	No	No	No	Yes	No	Yes	No	S	S	R	R	R	R	R	R	R	R	S	R	S	N
ABs52	Urine	Yes	Yes	Yes	No	Yes	No	No	No	R	R	R	R	R	R	S	S	R	R	S	R	S	Y
ABs53	Respiratory secretion	Yes	No	No	No	Yes	No	No	No	S	S	S	S	S	S	S	S	S	S	S	S	S	Y
ABs54	Respiratory secretion	Yes	Yes	Yes	No	Yes	No	No	No	R	R	R	R	R	R	S	S	R	R	S	R	S	Y
ABs55	Blood	Yes	Yes	Yes	No	Yes	No	No	No	R	R	R	R	R	R	S	S	R	I	S	R	S	Y
ABs56	Blood	No	Yes	Yes	No	Yes	No	No	No	NT	NT	R	R	R	R	S	S	R	NT	S	R	S	Y
ABs58	Urine	No	Yes	Yes	No	Yes	No	No	No	NT	NT	R	R	R	R	NT	R	R	NT	S	R	S	Y
ABs61	Blood	No	Yes	Yes	No	Yes	No	No	No	R	R	R	R	R	R	S	R	R	NT	S	NT	S	Y
ABs62	Catheter	No	No	No	No	Yes	Yes	No	No	NT	R	R	R	NT	S	S	S	R	R	NT	NT	NT	N
ABs66	Respiratory secretion	Yes	No	No	No	Yes	Yes	No	No	S	R	R	R	S	S	S	R	R	NT	S	NT	S	Y
ABs67	Catheter	Yes	Yes	Yes	No	Yes	Yes	No	No	I	R	R	R	R	R	S	I	R	NT	S	NT	S	Y
ABs71	Catheter	No	No	No	No	Yes	No	No	No	R	R	R	R	R	R	S	R	R	NT	S	NT	S	Y
ABs72	Respiratory secretion	Yes	No	No	No	Yes	No	No	No	I	R	R	R	R	R	S	R	R	NT	S	NT	S	N
ABs73	secreção ocular	Yes	No	No	No	Yes	No	No	No	S	S	R	S	S	S	R	R	R	R	NT	R	S	Y
ABs74	Blood	No	No	No	No	Yes	No	No	No	R	R	R	R	R	R	S	S	R	R	S	R	S	Y
ABs75	Catheter	Yes	No	No	No	Yes	No	No	No	S	S	S	S	S	S	NT	S	S	S	S	S	S	Y
ABs77	Respiratory secretion	Yes	No	No	No	Yes	Yes	No	No	R	R	R	R	R	R	S	R	R	NT	S	NT	S	Y
ABs78	Respiratory secretion	Yes	No	No	No	Yes	Yes	Yes	No	R	NT	R	R	R	R	R	R	R	NT	S	NT	S	Y
ABs80	Blood	Yes	Yes	Yes	No	Yes	Yes	No	No	R	NT	R	R	R	R	S	R	R	NT	S	NT	S	Y
ABs81	Respiratory secretion	Yes	Yes	Yes	No	Yes	No	No	No	R	R	R	R	R	R	R	R	R	R	S	R	S	Y
ABs88	Secreção de Skin	No	No	No	No		No	No	No	S	S	R	R	S	S	S	S	R	R	S	S	S	N
Total:																							
		43	32	29	0	55	17	2	0	34	39	54	52	43	43	12	32	51	20	5	24	4	45
		13	24	27	56	0	39	54	56	12	8	2	4	12	13	39	23	3	3	48	6	50	11
		56	56	56	56	55	56	56	56	46	47	56	56	55	56	51	55	54	23	53	30	54	56
